# Effect of an Ultra-Endurance Event on Cardiovascular Function and Cognitive Performance in Marathon Runners

**DOI:** 10.3389/fphys.2022.838704

**Published:** 2022-04-20

**Authors:** Andrew S. Perrotta, Andrew T. Jeklin, Shannon S. D. Bredin, Erin M. Shellington, Kai L. Kaufman, Amanda de Faye, Rosalin M. Miles, Darren E. R. Warburton

**Affiliations:** ^1^Department of Kinesiology, Langara College, Vancouver, BC, Canada; ^2^Department of Medicine, St. Vincent’s Hospital, Melbourne, VIC, Australia; ^3^Cardiovascular Physiology and Rehabilitation Laboratory, University of British Columbia, Vancouver, BC, Canada; ^4^Faculty of Education, University of British Columbia, Vancouver, BC, Canada; ^5^Cognitive and Motor Learning Laboratory, University of British Columbia, Vancouver, BC, Canada; ^6^Experimental Medicine Program, Faculty of Medicine, University of British Columbia, Vancouver, BC, Canada

**Keywords:** cardiovascular function, cognition, endurance exercise, ultra-marathon, race performance

## Abstract

**Background:**

Ultra-marathon running participation has become increasingly more popular in recent years; however, there is inconclusive evidence concerning the effects of participation on cognition and cardiovascular function. The purpose of this study was to examine alterations in cardiovascular function and cognitive performance and their association in ultra-marathon runners prior to and following an ultra-endurance event.

**Methods:**

In total, 24 runners (19 males and 5 females) participated in an ultra-marathon race (FatDog120) held in British Columbia, Canada. Participants competed in varying races distances [48 km (*n* = 2), 80 km (*n* = 7), 113 km (*n* = 3), and 193 km (*n* = 12)]. Cognition was assessed prior to and upon race completion using simple reaction time, choice reaction time, discrimination reaction time, and recognition memory (% correct). Cardiovascular function was assessed prior to and upon race completion using radial applanation tonometry for diastolic pulse contour examination.

**Results:**

Cognitive performance displayed significantly (*p* < 0.001) slower reaction times post-race for simple (30.2%), discrimination (22.7%), and choice reaction time (30.5%), as well as a significant (*p* < 0.05) reduction in memory test performance (−8.2%). A significant association between systemic vascular resistance and choice reaction time was observed post-race (*r* = 0.41, *p* < 0.05). Significant changes in post-race cardiovascular function were observed in resting heart rate (31.5%), cardiac output (27.5%), mean arterial blood pressure (−5.6%), total systemic resistance (−17.6%), systolic blood pressure (−7.0%), pulse pressure (−11.2%), and rate pressure product (22.4%). There was evidence of enhanced cardiovascular function being associated with improved cognitive performance before and after the ultra-endurance event.

**Conclusion:**

Ultra endurance running is associated with marked impairments in cognitive performance that are associated (at least in part) with changes in cardiovascular function in healthy adults.

## Introduction

Ultra-marathons are running events that extend beyond the traditional marathon length of 42.2 km. Depending on the event, this style of marathon may be limited to a fixed race time completion or by completing the required distance (irrespective of time). These events often involve changes in elevation, where participants are required to make multiple ascents and descents greater than 2,000 m while running over challenging terrain (Bonsignore et al., 2017).

Current systematic reviews examining the physiological and psychological demands of ultra-marathons have demonstrated the importance of elevated and sustained cardiovascular function and cognitive performance (Garbisu-Hualde and Santos-Concejero, 2020; Roebuck et al., 2018). This is important because ultra-marathon runners have indicated that both race strategy and race management are critical components for running a successful race (Simpson et al., 2014). Moreover, ultra-endurance athletes are often required to make critical decisions while navigating potentially life-threatening terrain and environmental conditions. As such, the necessity for sustained cognitive performance throughout an ultra-endurance event is required to support athletes in critical decision making at any period of the race. Limited research has been conducted in this field. However, Hurdiel et al. (2015) demonstrated a significant impairment in post-race cognitive function when compared to pre-race in ultra-marathon runners that occurred irrespective of race length. Ultra-marathons continue to attract experienced marathon runners of all ages seeking new experiences in testing their physical and mental capacity. Recent evidence has shown that speed of processing and decision making, measured by simple and choice reaction times, becomes more variable through adulthood and decreases linearly at the age of 50 (Der and Deary, 2006). This is particularly salient given the average ages of participants engaging in ultra-endurance events and the large percentage of runners who are over the age of 50 (Nikolaidis and Knechtle, 2018).

It is well known that regular exercise is an effective primary and secondary preventative strategy for at least 25 chronic medical conditions (Warburton et al., 2006). Moreover, physical activity and/or exercise have been shown to be protective against cognitive decline in aging populations (Barnes et al., 2003). However, continued research has demonstrated the potential adverse effects of extreme exercise, such as the effect of ultra-endurance events on cardiovascular function (Burr et al., 2012; Bonsignore et al., 2017) and acute measures of cognition (Hurdiel et al., 2015). For example, there is evidence that participation in ultra-marathon events may lead to an acute and transient increase in resting arterial stiffness (Burr et al., 2014), impaired left ventricular systolic and diastolic function (Burr et al., 2012), a reduction in large artery compliance (Bonsignore et al., 2017), and increased reaction time with the greatest decrements occurring when performing the most complex cognitive task (Burrows et al., 2014). Given the important health- and performance-related consequences of cardiovascular and cognitive function, this research has important implications for individuals that engage routinely in this form of extreme exercise (ultra-endurance events). The potential for cognitive and cardiovascular impairment during and following ultra-endurance events, and a paucity of studies understanding cardiovascular and cognitive function need to be reconciled to understand the overall health implications of ultra-endurance events.

Accordingly, the purposes of this investigation were to determine: (1) if cognitive and cardiovascular function are reduced following an ultra-endurance event, and (2) the association between cardiovascular and cognitive function following an ultra-endurance event. We hypothesized that there would be a significant association between cognitive performance and cardiovascular function following an ultra-endurance event and cognitive performance (as measured primarily by various reaction time tasks) would be slower post-race compared to pre-race.

## Materials and Methods

### Participants

A total of 24 experienced ultra-marathon runners, who were registered in an ultra-endurance trail race, were recruited and provided written informed consent to participate in the research. Participant characteristics are provided in Table 1. Each participant was recruited 1 day preceding the start of the event. Participants completed the Physical Activity Readiness Questionnaire for Everyone (PAR-Q+) to ensure they were ready to participate in physical activity (Warburton et al., 2011). Each participant self-reported they had not smoked nor participated in strenuous exercise 24-h prior to baseline testing. In addition, each participant confirmed through self-report that they had refrained from any form of caffeine from 2 h prior, and had not consumed alcohol from 12-h prior to baseline testing. This investigation was approved by and executed in exact accordance with the Clinical Research Ethics Board at the University of British Columbia in accordance with the ethical standards established by Declaration of Helsinki and its later amendments.

**TABLE 1:** Participant characteristics.

Participants			Age	Weight	Height	BMI	Waist circumference	Body fat	Ultra-marathon experience	Mean weekly training distance
								
Male	Female	(*n*)	(years)	(kg)	(cm)	(kg⋅m^–2^)	(cm)	(%)	(years)	(km)
19	5	24	38.6 ± 9.8	70.8 ± 9.8	174.2 ± 9.0	23.2 ± 1.9	81.6 ± 7.2	17.5 ± 5.7	4.0 ± 1.0	87.0 ± 1.0

*Values are displayed as a mean (±SD).*

### Study Design

This was an observational study that examined cardiovascular function and cognitive performance prior to and upon completion of an ultra-marathon and their associations in marathon runners. Race distances were: 30 miles (48.3 km), 50 miles (80.5 km), 70 miles (112.7 km), and 120 miles (193.1 km). The ultra-endurance (FatDog120) event was held in the Cascade Mountains in southwestern British Columbia in mid-August with an average temperature of 17.5°C (range 8.7–26.3°C). The race course included a total elevation gain of 3,276 m in the 80.5-km event and 8,673 m in the 193.1-km event. The highest ascent on the 195-km course was 2,300 m and the lowest ascent was 600 m. The highest ascent on the 48.3- and 80.5-km course was 2,200 m and the lowest ascent was 500 m (Bonsignore et al., 2017). The challenging terrain for each event included technical climbs and non-technical flat trails. Nutrition and hydration throughout the study period was at *ad libitum* at the discretion of each runner. Baseline measures for participants were assessed 24-h before the start of the race and within 45-min of completing the ultra-marathon. Both pre- and post-race assessments were conducted in a controlled thermoneutral environment (located immediately adjacent to the race start/finish line).

### Anthropometric Measures

Anthropometric assessments were recorded without shoes. Body weight and body composition was examined via bioelectrical impedance (Tanita Model TBF 300A, Arlington Heights, IL, United States) utilizing a single frequency analysis for estimating body fat percentage. Height (cm) and weight (kg) were used to calculate the body mass index of each participant (kg⋅m^–2^).

### Cognitive Measures

Cognition, more specifically, speed of processing, decision making, and recognition memory, was assessed using four tasks: simple reaction time (ms), choice reaction time (CRT; ms), discrimination reaction time (ms), and a recognition memory test (% correct). Tasks were administered using E-prime software Version 3.0 (Psychology Software Tools, Sharpsburg, PA, United States) on an ASUS tablet (AsusTek, Taipei, Taiwan). All tasks were supervised by researchers and participants received both an instruction screen and had three practice trials for each task prior to testing. Each reaction time task consisted of 20 stimuli that appeared at random inter-stimulus intervals between 500 and 2,500 ms. For simple reaction time, participants were shown a stimulus (blue dot) on a screen and instructed to respond as fast as possible by pressing the “v” key (which also had a blue dot placed on the key for reference). For discrimination reaction time (also referred to in the literature as recognition reaction time or go/no-go reaction time), participants were randomly shown a blue, green, or red stimulus (dot on the screen) and instructed to respond only to the green dot by pressing the “n” key as fast as possible. In choice reaction time, participants were randomly shown a blue or green stimulus and instructed to respond as fast as possible by pressing the “v” key when the blue stimulus appeared on the screen or the “n” key when the green stimulus appeared on the screen. During the recognition memory task, participants observed a group of playing cards that were shown on the screen for a total of 4 s. Following this period, a single card appeared on the screen in which participants were instructed to respond as quickly as possible if the single card was previously shown in the group of playing cards. Participants responded by pressing the “v” key if the card was previously shown in the group of playing cards or the “n” key if the card was not previously shown. Data were time and date stamped and manually extracted from E-prime for analysis.

### Cardiovascular Measures

After a 10-min stabilization period, cardiovascular assessments were conducted laying in a supine position while blindfolded and wearing noise canceling ear protection in a thermoneutral environment. All cardiovascular measures were conducted three times with the mean value used for analysis. Heart rate (bpm) and blood pressure (mmHg) were recorded using an automated blood pressure cuff monitor (BPM-100, BpTru^®^, VSM MedTech Ltd, BC, Canada) positioned over the brachial artery of the left arm. Pulse pressure (mmHg), rate pressure product (mmHg.bpm), and mean arterial pressure (mmHg) were calculated. Applanation tonometry (HDI/Pulsewave CR-2000) (Hypertension Diagnostics Inc., Eagan, MN, United States) was used to non-invasively measure small (C_2_), large (C_1_) arterial compliance, and total systemic vascular resistance (Saugel et al., 2015). Participants were instructed to lie quietly with the right wrist constrained with a wrist stabilizer in the effort to enhance the signal quality of radial artery pulse-waves. After palpation, the sensor was positioned over the area of maximal radial artery pulsation. Blood pressure indices were recorded using an automated blood pressure cuff positioned on the upper extremity of the arm. Change in right arterial waveforms were examined over a 30-s period. Indices of cardiac function such as, cardiac output (L⋅min^−1^), stroke volume (mL⋅beat^−1^) and cardiac index (L⋅min^−1^⋅m^−2^) were concurrently estimated. The standardization from utilizing automated measurement techniques required minimal skill from the technician, thereby enhancing the reliability in measures in healthy participants while yielding a high accuracy (Zimlichman et al., 2005).

### Data Analysis

Data was analyzed using Microsoft^®^ EXLSTAT 5.1. All data in text, tables and figures are displayed as means (±SD). Normality of data sets were examined using a Shapiro-Wilk’s test for normality (*p* > 0.05). A two-tailed paired sample t-test was utilized to examine variation in pre-and post-race cardiovascular and cognitive measures. Significance was declared using a probability of *p* < 0.05. A Pearson Product correlation coefficient (*r*) was employed to examine the strength of association between each cardiovascular and cognitive performance measure during the pre-race and post-race time period. The following principles were applied for identifying the strength of correlation; 0.10–0.29 small, 0.30–0.49 moderate, and >0.50 large (Cohen, 1988).

## Results

Cardiac output (and index) was significantly increased post-race as a result of significant increases in resting heart rate (31.5%) with a small non-significant decrease in stroke volume post-race (−4.8%, *p* = 0.28; Table 2). There were significant reductions in systolic blood pressure (−7.0%), diastolic pressure (−4.4%), mean arterial blood pressure (−5.6%), pulse pressure (−11.2%), and systemic vascular resistance (−17.6%; Table 2). Rate pressure product was significantly increased (22.4%) following the race. Large arterial compliance (8.0%, *p* = 0.25) and small arterial compliance (−6.8%, *p* = 0.69) were not statistically changed following the ultra-endurance event (Table 3).

**TABLE 2:** Pre- and post-race resting cardiac function.

Variable	Pre-race	Post-race	% Change	*p*
Resting heart rate (bpm)	61.4 ± 8.6	80.7 ± 13.3	31.5%	0.00
193 km (*n* = 12)	60.4 ± 10.6	77.1 ± 13.8	27.6%	
113 km (*n* = 3)	67.3 ± 2.3	82.7 ± 3.2	22.8%	
80 km (*n* = 7)	62.5 ± 5.1	87.6 ± 15.1	40.1%	
48 km (*n* = 2)	53.8 ± 5.9	74.7 ± 5.7	38.7%	
Cardiac output (L⋅min^–1^)	4.1 ± 0.82	5.3 ± 1.3	27.5%	0.00
193 km (*n* = 12)	4.4 ± 0.57	5.2 ± 1.4	17.2%	
113 km (*n* = 3)	3.4 ± 1.3	4.8 ± 0.19	42.2%	
80 km (*n* = 7)	4.2 ± 0.45	5.7 ± 1.5	34.8%	
48 km (*n* = 2)	3.0 ± 1.4	4.7 ± 0.24	58.1%	
Stroke volume (mL⋅beat^−1^)	68.1 ± 15.3	64.8 ± 7.5	−4.8%	0.28
193 km (*n* = 12)	74.6 ± 12.1	66.9 ± 8.3	−10.3%	
113 km (*n* = 3)	50.2 ± 19.6	57.9 ± 1.5	15.4%	
80 km (*n* = 7)	67.7 ± 5.5	64.5 ± 6.6	−4.7%	
48 km (*n* = 2)	57.1 ± 33.1	63.3 ± 8.1	10.9%	
Cardiac index (L⋅min^–1^⋅m^–2^)	2.3 ± 0.52	2.8 ± 0.64	22.1%	0.00
193 km (*n* = 12)	2.4 ± 0.34	2.6 ± 0.46	7.2%	
113 km (*n* = 3)	1.87 ± 0.79	2.64 ± 0.25	41.3%	
80 km (*n* = 7)	2.5 ± 0.35	3.3 ± 0.84	34.1%	
48 km (*n* = 2)	1.5 ± 0.62	2.34 ± 0.03	60.8%	

*Values displayed as means (±SD).*

**TABLE 3:** Pre- and post-race vascular function.

Variable	Pre-race	Post-race	% Change	*p*
Systolic blood pressure (mmHg)	120.2 ± 16.6	111.7 ± 9.6	−7.0%	0.02
193 km (*n* = 12)	121.3 ± 1 2.0	112.6 ± 10.8	−7.2%	
113 km (*n* = 3)	105.3 ± 1 9.9	105.1 ± 8.6	−0.2%	
80 km (*n* = 7)	125.9 ± 17.6	112.2 ± 9.4	−10.9%	
48 km (*n* = 2)	115.5 ± 34.2	114.5 ± 1.2	−0.9%	
Diastolic blood pressure (mmHg)	74.2 ± 9.0	70.9 ± 6.2	−4.4%	0.06
193 km (*n* = 12)	71.8 ± 8.6	70.5 ± 7.6	−1.8%	
113 km (*n* = 3)	73.4 ± 6.0	70.1 ± 5.8	−4.5%	
80 km (*n* = 7)	78.3 ± 11.4	71.3 ± 5.3	−8.9%	
48 km (*n* = 2)	75.7 ± 4.7	73.5 ± 3.5	−2.9%	
Mean arterial pressure (mmHg)	89.5 ± 10.7	84.5 ± 7.0	−5.6%	0.02
193 km (*n* = 12)	88.3 ± 9.0	84.5 ± 8.4	−4.3%	
113 km (*n* = 3)	84.1 ± 10.1	81.8 ± 6.7	−2.7%	
80 km (*n* = 7)	94.2 ± 13.4	84.9 ± 6.3	−9.8%	
48 km (*n* = 2)	88.9 ± 14.5	87.2 ± 2.0	−2.0%	
Pulse pressure (mmHg)	46.0 ± 11.9	40.8 ± 5.6	−11.2%	0.04
193 km (*n* = 12)	49.5 ± 8.3	42.1 ± 5.7	−14.9%	
113 km (*n* = 3)	31.9 ± 15.5	35.0 ± 2.9	9.8%	
80 km (*n* = 7)	47.6 ± 7.3	40.9 ± 5.9	−14.1%	
48 km (*n* = 2)	39.8 ± 29.5	41.0 ± 4.7	2.9%	
Total systemic resistance (dyne.s.cm^–5^)	1,365.5 ± 180.4	1,124.8 ± 239.3	−17.6%	0.00
193 km (*n* = 12)	1,379.0 ± 199.0	1,177.3 ± 261.6	−14.6%	
113 km (*n* = 3)	1,259.0 ± 244.3	913.7 ± 140.3	−27.4%	
80 km (*n* = 7)	1,387.3 ± 126.2	1,150.6 ± 229.9	−17.1%	
48 km (*n* = 2)	1,368.5 ± 241.1	1,037.0 ± 144.2	−24.2%	
Rate pressure product (mmHg.bpm)	7,357.9 ± 1392.4	9,006.7 ± 1720.4	22.4%	0.00
193 km (*n* = 12)	7,326.5 ± 1444.4	8,618.6 ± 1356.4	17.6%	
113 km (*n* = 3)	7,067.4 ± 1130.7	8,706.9 ± 1040.2	23.2%	
80 km (*n* = 7)	7,890.7 ± 1438.1	9,932.1 ± 2478.8	25.9%	
48 km (*n* = 2)	6,117.1 ± 1159.3	8,546.0 ± 559.7	39.7%	
Large arterial compliance (mL.mmHg^–1^ × 10)	17.6 ± 2.5	19.1 ± 6.3	8.0%	0.25
193 km (*n* = 12)	17.9 ± 4.4	19.4 ± 5.5	8.2%	
113 km (*n* = 3)	18.3 ± 0.6	24.9 ± 9.2	36.5%	
80 km (*n* = 7)	16.9 ± 3.9	15.2 ± 4.6	−10.3%	
48 km (*n* = 2)	17.5 ± 1.2	21.7 ± 7.2	24.4%	
Small arterial compliance (mL.mmHg^–1^ × 100)	9.0 ± 2.4	8.4 ± 5.6	−6.8%	0.69
193 km (*n* = 12)	9.0 ± 2.6	9.1 ± 5.7	1.8%	
113 km (*n* = 3)	9.6 ± 1.1	12.9 ± 9.9	34.3%	
80 km (*n* = 7)	8.6 ± 2.5	5.5 ± 2.7	−35.9%	
48 km (*n* = 2)	10.0 ± 4.0	8.0 ± 1.8	−20.5%	

*Values displayed as means (±SD).*

**FIGURE 1:**
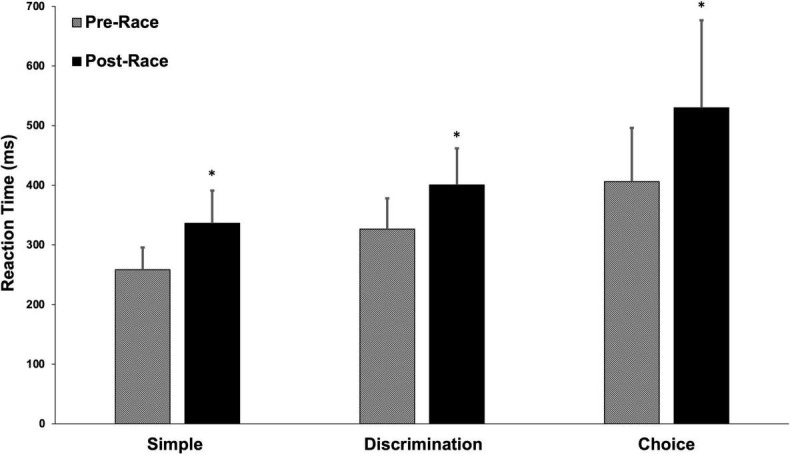
Pre- and post-race reaction time. Values are displayed as a mean (±SD). *p < 0.0001.

Pre- and post-race cognitive performance measures are illustrated in Figure 1. Post-race simple reaction time was slower by 30.2% (258.3 ± 37.5 vs. 336.3 ± 54.8 ms, *p* < 0.001). Post-race discrimination reaction time was slower by 22.7% (326.6 ± 51.5 vs. 400.8 ± 61.1 ms, *p* < 0.001) and choice reaction time was slower by 30.5% (406.0 ± 90.1 vs 530.0 ± 146.6 ms, *p* < 0.001). Recognition memory performance (% correct), deteriorated by 8.2% post-race (88.9 ± 8.7 vs. 81.6 ± 13.5%; *p* < 0.05). The association between indices of cognitive performance and cardiovascular function are displayed as a correlation matrix with heat map for both the pre-race period (Table 4) and post-race period (Table 5). Pre-race, the strongest relationships were seen between cognitive reaction time measures (i.e., simple, discrimination reaction time, and choice reaction time) and total systemic resistance and heart rate (Table 4). Post-race, the strongest relationship was observed between choice reaction time and systemic vascular resistance (*r* = 0.41).

**TABLE 4:** Correlation matrix: pre-race associations between indices of cardiovascular function and cognitive performance and displayed as Pearson correlation coefficient.

Variable	Cardiovascular measure												Cognitive measure			
	1	2	3	4	5	6	7	8	9	10	11	12	13	14	15	16
1. Systolic Blood Pressure (mmHg)	**1**															
2. Diastolic Blood Pressure (mmHg)	0.74*	**1**														
3. Mean Arterial Pressure (mmHg)	0.92*	0.94*	**1**													
4. Pulse Pressure (mmHg)	0.83*	0.24	0.55*	**1**												
5. Total Systemic Resistance (dyne⋅sec⋅cm^−5^)	0.34	0.1	0.23	0.40*	**1**											
6. Rate Pressure Product (mmHg⋅bpm)	0.61*	0.78*	0.75*	0.24	−0.09	**1**										
7. Large Arterial Compliance (mL⋅mmHg^−1^× 10)	0.11	0	0.06	0.16	0.28	−0.05	**1**									
8. Small Arterial Compliance (mL⋅mmHg^−1^× 100)	0.27	0.13	0.21	0.29	0	0.15	0.35	**1**								
9. Heart Rate (bpm)	−0.19	0.27	0.06	−0.50*	−0.44*	0.66*	−0.17	−0.07	**1**							
10. Stroke Volume (mL⋅beat^1^)	0.43*	0.01	0.22	0.61*	0.04	0.52*	0.05	0.24	0.22	**1**						
11. Cardiac Output (L⋅min^−1^)	0.44*	−0.27	0.07	0.87*	0.35	−0.14	0.15	0.22	−0.62*	0.62*	**1**					
12. Cardiac Index (L⋅min^−1^⋅m^−2^)	0.18	−0.12	0.02	0.36	0.13	0.39	−0.01	0.19	0.3	0.85*	0.44*	**1**				
13. Simple Reaction (ms)	0.06	−0.13	−0.05	0.2	0.39	−0.23	−0.1	0.1	−0.35	0.03	0.26	0.12	**1**			
14. Discrimination Reaction Time (ms)	0.16	−0.14	0	0.35	0.50*	−0.19	0.24	0.09	−0.41*	0.13	0.41*	0.18	0.70*	**1**		
15. Choice Reaction Time (ms)	0.08	−0.08	0	0.18	0.56*	−0.23	0.39	0.13	−0.38	−0.04	0.23	0	0.67*	0.69*	**1**	
16. Memory (% Correct)	−0.37	−0.05	−0.21	−0.50*	−0.19	−0.19	−0.17	−0.3	0.13	−0.44*	−0.47*	−0.24	−0.32	−0.47*	−0.36	**1**

*p < 0.05.

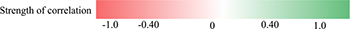

**TABLE 5:** Correlation matrix: post-race associations between indices of cardiovascular function and cognitive performance and displayed as Pearson correlation coefficient.

Variable	Cardiovascular measure												Cognitive measure			
	1	2	3	4	5	6	7	8	9	10	11	12	13	14	15	16
1. Systolic Blood Pressure (mmHg)	1															
2. Diastolic Blood Pressure (mmHg)	0.82*	1														
3. Mean Arterial Pressure (mmHg)	0.94*	0.97*	1													
4. Pulse Pressure (mmHg)	0.78*	0.28	0.52*	1												
5. Total Systemic Resistance (dyne⋅sec⋅cm^−5^)	0.24	0.29	0.28	0.08	1											
6. Rate Pressure Product (mmHg⋅bpm)	0.39	0.2	0.3	0.44*	−0.2	1										
7. Large Arterial Compliance (mL⋅mmHg^−1^× 10)	−0.04	−0.14	−0.1	0.08	0.05	0.34	1									
8. Small Arterial Compliance (mL⋅mmHg^−1^× 100)	−0.35	−0.3	−0.34	−0.25	0.19	−0.44*	0.02	1								
9. Heart Rate (bpm)	−0.03	−0.16	−0.11	0.12	−0.36	0.90*	0.36	−0.35	1							
10. Stroke Volume (mL⋅beat^1^)	0.08	−0.33	−0.16	0.50*	−0.33	0.81*	0.38	−0.28	0.85*	1						
11. Cardiac Output (L⋅min^−1^)	0.23	−0.36	−0.11	0.79*	−0.13	0.29	0.16	−0.06	0.23	0.70*	1					
12. Cardiac Index (L⋅min^−1^. m^−2^)	−0.02	−0.34	−0.21	0.33	−0.13	0.77*	0.34	−0.25	0.86*	0.93*	0.54*	1				
13. Simple Reaction (ms)	−0.28	−0.24	−0.27	−0.22	0.13	−0.18	0.14	−0.14	−0.07	−0.1	−0.08	−0.11	1			
14. Discrimination Reaction Time (ms)	rgb] .996, .933, .933−0.1	−0.01	−0.05	−0.15	0.36	−0.35	−0.25	−0.05	−0.33	−0.34	−0.15	−0.28	0.72*	1		
15. Choice Reaction Time (ms)	−0.29	−0.09	−0.19	−0.37	0.41*	−0.35	−0.06	−0.12	−0.24	−0.34	−0.31	−0.2	0.77*	0.78*	1	
16. Memory (% Correct)	−0.03	0.03	0.01	−0.09	−0.19	0.08	0.08	−0.08	0.1	0.05	−0.09	0.14	−0.42*	−0.44*	−0.18	1

*p < 0.05.

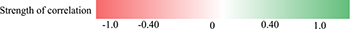

## Discussion

The purpose of this investigation was to examine cognitive performance and cardiovascular function prior to and upon completion of an ultra-endurance event (i.e., the Fatdog 120). The main findings of this study were as follows: (1) cognitive performance was significantly diminished post-race, and (2) cardiovascular function displayed significant associations with indices of cognitive performance during both the pre-race and post-race period, with total systemic resistance and heart rate exhibiting the strongest relationships with measures of reaction time at baseline (i.e., simple reaction time, discrimination reaction time, and choice reaction time), and total systemic vascular resistance displaying the strongest association with cognitive performance (i.e., choice reaction time) during the post-race period. Our findings support and extend the work of Hurdiel et al. (2015) demonstrating significantly slower reaction times for simple reaction time, discrimination reaction time, and choice reaction time along with a significant reduction in memory test performance after an ultra-endurance event.

Our current study revealed important insight into the effects of prolonged strenuous exercise on arterial compliance. There is inconclusive evidence concerning the effects of participation in ultra-endurance events. Some studies have revealed reduced measures of arterial compliance (or increased arterial stiffness) after prolonged strenuous exercise (Burr et al., 2012, 2014; Bonsignore et al., 2017), while others have revealed no significant changes (Vlachopoulos et al., 2010). Our current findings revealed no significant change in arterial compliance as assessed by applanation tonometry. This is contrary to previous findings wherein increased arterial stiffness (Burr et al., 2014) and reduced arterial compliance (Burr et al., 2012; Bonsignore et al., 2017) were observed after an ultra-endurance event. However, our findings are consistent with those of others (Vlachopoulos et al., 2010).

Importantly, in the current investigation markers of systemic vascular resistance were also decreased alongside other markers supporting post-exercise hypotension (i.e., reduced systolic and diastolic blood pressure). It is plausible that the unchanged arterial compliance was the result of the post-exercise hypotension observed in our trial (Vlachopoulos et al., 2010; Tomoto et al., 2015) or the effect of exercise-related systemic inflammation (Tomoto et al., 2015).

The majority of literature in the area has focused on shorter duration aerobic exercise in well controlled laboratory conditions. Cardiac parasympathetic reactivation following short-term aerobic exercise is well-established (Stanley et al., 2013). Stanley et al. (2013) have argued that metaboreflex stimulation (e.g., muscle and blood acidosis) is a key determinant of parasympathetic reactivation seen shortly post-exercise (e.g., 0–90 min). Moreover, they argue that time for complete cardiac autonomic recovery following high-intensity exercise is at least 48 h. However, there is comparatively limited evidence related to sympathetic and parasympathetic control/balance following prolonged strenuous exercise (in particular ultra-endurance events) (Hautala et al., 2001; Scott et al., 2009; Foulds et al., 2014; Martínez-Navarro et al., 2019). Although limited in number, there is compelling evidence of blunted parasympathetic activity following prolonged ultra-endurance events requiring considerable time for full cardiac autonomic recovery. For instance, Hautala et al. (2001) revealed that cardiac vagal outflow (as assessed by heart rate variability) was blunted for several hours following prolonged (75 km) vigorous cross-country skiing race (Hautala et al., 2001). Previously, we (Foulds et al., 2014) revealed that recreational ultra-endurance athletes demonstrated significantly greater sympathetic modulation and lower parasympathetic modulation (reflecting increased sympathovagal balance) following an ultra-endurance mountain event (involving 120 and 195 km race lengths). Martínez-Navarro et al. (2019) revealed that vagally-mediated heart rate variability was decreased and cardiac autonomic modulation was less complex following a 118 km mountain ultra-marathon. Similar to our current findings, a significant increase in heart rate (58.8 ± 7.9 vs 76.3 ± 9.6 bpm, respectively) was also observed following the ultra-endurance event (Martínez-Navarro et al., 2019). These authors also revealed that the athletes with the higher baseline overall and vagally-mediated heart rate variability achieved faster race finishing times. A recent study by Swart and Constantinou (2020) revealed that ultra-endurance mountain bike participation led to diminished vagal activity (evaluated by heart rate variability) and a shift to sympathetic dominance (Swart and Constantinou, 2020). Collectively, our current findings and that of several other studies support the hypothesis that ultra-endurance events can lead to a transient shift to sympathetic dominance. However, this finding may not be uniform as Scott et al. (2009) revealed no significant changes in autonomic function (as evaluated by heart rate variability) following a 160 km ultra-endurance trail run (Scott et al., 2009).

The increase in heart rate in the face of a reduction in systemic vascular resistance and relative modest post-exercise hypotension reflects the complex adjustments in cardiovascular control and the potential interplay of several factors (MacDonald, 2002; Halliwill et al., 2014; Romero et al., 2017). As reviewed by Romero et al. (2017), arterial blood pressure is the product of cardiac output (arterial inflow) divided by systemic vascular conductance [inverse of systematic vascular resistance (often referred to as total peripheral resistance)] (Romero et al., 2017). In our current study, there was a significant increase in cardiac output (mediated by an increase in heart rate). Systematic vascular resistance is determined by the level of vasoconstriction or vasodilation of vascular beds (Romero et al., 2017). Key potential mechanisms underlying the modest post-exercise hypotension observed in our study include baroreflex and thermoreflex resetting, vasodilatation (dependent upon the activation of histamine H_1_- and H_2_-receptors), and pre-synaptic inhibition of sympathetic vasoconstrictor nerves (Halliwill et al., 2014; Romero et al., 2017). Other factors that likely affect post-exercise hypotension include fluid/blood volume status, ambient heat, and orthostatic stress (Halliwill et al., 2014). However, the small non-significant decrease in both stroke volume and body weight suggests that marked hypovolemia and a reduction in venous return were likely absent following the event.

Physiological measures such as maximal aerobic power and its determinants (in particular cardiac function) have shown to be important factors in race completion time (Davies and Thompson, 1979; Gledhill et al., 1999). High cardiorespiratory fitness has also been associated with improved cognitive performance measures (such as attention and memory) (Hillman et al., 2008). This association in ultra-marathon runners has most recently been demonstrated by Cona et al. (2015) where runners with faster race completion times displayed better inhibitory control than slower runners supporting the role of fitness level on cognitive performance. The contextual relationship between cerebral blood flow and cognitive function has been well reviewed (Ogoh and Tarumi, 2019). Cerebral blood flow has shown to be well maintained with minimal changes in perfusion irrespective of significant alterations in mean arterial blood pressure (Lassen, 1959). This phenomenon has been characterized as cerebral autoregulation, a homeostatic mechanism that regulates cerebral blood flow to maintain cerebral perfusion, using alterations in vasomotor response to control cerebral vascular resistance (Aaslid et al., 1989). Current reviews around the relationship between arterial blood pressure and cerebral blood flow have emphasized the complicated nature between each regulatory mechanism; however, there remains compelling evidence for both direct and indirect effects of arterial blood pressure on cerebral blood flow regulation (Ogoh and Tarumi, 2019). Specifically, cardiac output is thought to directly influence cerebral blood flow in healthy individuals during exercise and rest, with the latter demonstrating a greater association (Ogoh et al., 2005), suggesting enhanced cardiac function would potentially improve cognitive performance. Furthermore, cerebral vascular perfusion may be independent of cardiac output as long as blood pressure remains stable (Van Lieshout et al., 2003).

In our current study, we examined resting measures of cardiovascular function and the association with markers of cognitive performance. There was evidence of enhanced cardiovascular function being associated with improved cognitive performance at rest and after an ultra-endurance event. We hypothesize that the redistribution of cardiac output to skeletal muscle as a response to the metaboreflex for meeting metabolic demands (Laughlin et al., 2011), in addition to various physiological responses causing post-exercise hypotension (MacDonald, 2002), may have sufficiently altered cerebral vascular blood flow thereby influencing and decreasing cognitive performance (Van Lieshout et al., 2003). This, however, was likely tempered by the global increase in cardiac function (particularly heart rate) potentially attenuating the decline observed in cognitive performance. Further research in this area is warranted to fully elucidate the mechanisms responsible for our findings.

The immediate response after completing an ultra-marathon involves alterations in energy balance, increased hormone secretion, musculoskeletal damage, and/or immunosuppression (Knechtle and Nikolaidis, 2018). It is not fully clear how this multifaceted response to exercise affects cognitive function. However, the lack of association between several of the indices of cognition and cardiovascular function in the current study may have been the result of complex physiological mechanisms occurring post-race. It is also plausible that the age of our participants (37.0 ± 9.7 y) may have influenced our cognitive findings. Cognitive measures (such as choice and simple reaction time) have been shown to decrease significantly after the age of 50 (Der and Deary, 2006). It is plausible that our findings may have been accentuated in older participants. Further research is clearly warranted owing to the increasing participation rates in older adults within ultra-endurance events (Scheer, 2019). Age and sex are becoming increasingly known factors influencing ultra-marathon race performance. For instance, a recent study showed that sex differences were attenuated in ultra-endurance performance with increasing distance and age (Waldvogel et al., 2019). The participants in our study were around 20% female. Females have been shown to have slower and more accurate reaction time (Der and Deary, 2006; Dykiert et al., 2012). Further research should examine the potential influences of sex (Scheer, 2019).

Additionally, we suggest that the reduced cognitive performance seen in our study may have been from a combination of physical and mental fatigue, as well as extended wakefulness. Many participants in our study had been awake for >24 h and completed the race, and subsequent cognitive testing, in the early morning hours (between 12 and 6 a.m.). This is a well-established time when cognitive performance reaches a nadir (Durmer and Dinges, 2005; Van Dongen and Dinges, 2005). Performance decrements on cognitive testing have shown to be greatest in the early morning hours following an ultra-endurance event (Doppelmayr et al., 2005; Hurdiel et al., 2018). Owing to the influence of extreme physical and psychological demands of participating in an ultra-marathon event, we expected cognitive performance would decrease post-race. The current study contradicts previous work displaying prolonged physical fatigue may in fact result in a reduction of psychomotor and neuromuscular function; however, it does not necessarily have to be associated with mental fatigue and a reduction in cognitive functioning (Wollseiffen et al., 2016; Krokosz et al., 2020). These studies showed no significant change in cognitive performance following prolonged exercise suggesting that enhanced physical conditioning may have an impact on the cognitive performance post-race; however, further empirical evidence is required.

## Limitations

A potential limitation in the current study was the absence of measures of blood volume that may have accounted for the reduction in stroke volume and blood pressure. However, the non-significant reduction in post-race body weight (kg) (−0.8%, *p* = 0.10) is suggestive that hypovolemia was likely absent, as ultra-marathon runners are habitually trained to consume water *ad libitum* in an effort to maintain total body water, hematocrit, and hemoglobin (Robach et al., 2014; Ramos-Campo et al., 2016; Belinchon-deMiguel and Clemente-Suarez, 2018).

We recognize that the small sample size in each race length limited our ability to examine more closely the temporal changes that occur with both cardiovascular function and cognitive performance across different race lengths. Furthermore, participant recruitment was controlled by the availability and desire of registered runners. This may have resulted in a selection bias, affecting the findings of this study. Further research with larger sample sizes is warranted in this area to fully elucidate the effects of race distance on cardiovascular function and cognitive performance.

We acknowledge that cerebral vascular blood flow was not examined in the current study, and as such, we encourage further inquiry into the association between cerebral blood flow and cognitive performance after completing an ultra-endurance event to expand on our current findings. We also encourage future studies to include ultra-endurance athletes > 50 years of age, as they represent an increasing demographic in the sport and may provide further insight into age, cognition, and prolonged exercise.

## Conclusion

This study revealed significant reductions in cardiovascular function and cognitive performance in healthy runners after completing an ultra-marathon event. There were additional associations between indices of cognitive performance and cardiovascular function during the pre-race period when compared to the post-race period. This study revealed emerging evidence that systemic vascular resistance is inversely associated with cognitive performance prior to and upon completing an ultra-endurance event. It remains unclear the exact mechanisms responsible for these findings. Future research is recommended to examine the association between cerebral blood flow and cognitive performance after performing an ultra-endurance event.

## Data Availability Statement

The raw data supporting the conclusions of this article will be made available by the authors, without undue reservation.

## Ethics Statement

The studies involving human participants were reviewed and approved by Clinical Research Ethics Board at the University of British Columbia. The patients/participants provided their written informed consent to participate in this study.

## Author Contributions

SB and DW contributed to conception, design, implementation of the study and secured funding for the study. KK, SB, and DW organized the race event and related research study. AP performed the statistical analyses. AJ, AP, and SB wrote the first draft of the manuscript. SB, AP, AJ, ES, and DW wrote sections of the manuscript. RM served as the first nations advisor for this research. All authors contributed to the revision of the manuscript and approved the final submitted version.

## Conflict of Interest

The authors declare that the research was conducted in the absence of any commercial or financial relationships that could be construed as a potential conflict of interest.

## Publisher’s Note

All claims expressed in this article are solely those of the authors and do not necessarily represent those of their affiliated organizations, or those of the publisher, the editors and the reviewers. Any product that may be evaluated in this article, or claim that may be made by its manufacturer, is not guaranteed or endorsed by the publisher.

## References

[B1] Aaslid R. Lindegaard K. F. Sorteberg W. Nornes H. (1989). Cerebral autoregulation dynamics in humans. *Stroke* 20 45–52. 10.1161/01.str.20.1.45 2492126

[B2] Barnes D. E. Yaffe K. Satariano W. A. Tager I. B. (2003). A longitudinal study of cardiorespiratory fitness and cognitive function in healthy older adults. *J. Am. Geriatr. Soc.* 51 459–465. 10.1046/j.1532-5415.2003.51153.x 12657064

[B3] Belinchon-deMiguel P. Clemente-Suarez V. J. (2018). Psychophysiological, Body Composition, Biomechanical and Autonomic Modulation Analysis Procedures in an Ultraendurance Mountain Race. *J. Med. Syst.* 42:32. 10.1007/s10916-017-0889-y 29305660

[B4] Bonsignore A. Bredin S. S. D. Wollmann H. Morrison B. Jeklin A. Buschmann L. (2017). The influence of race length on arterial compliance following an ultra-endurance marathon. *Eur. J. Sport Sci.* 17 441–446. 10.1080/17461391.2016.1262453 27923330

[B5] Burr J. F. Bredin S. S. Phillips A. Foulds H. Cote A. Charlesworth S. (2012). Systemic arterial compliance following ultra-marathon. *Int. J. Sports Med.* 33 224–229. 10.1055/s-0031-1297956 22261822

[B6] Burr J. F. Phillips A. A. Drury T. C. Ivey A. C. Warburton D. E. (2014). Temporal response of arterial stiffness to ultra-marathon. *Int. J. Sports Med.* 35 658–663. 10.1055/s-0033-1358478 24408767

[B7] Burrows E. J. Warburton D. E. Bonsignore A. Buschmann L. Robertson J. Thickson W. (2014). Cognitive Function Following an Ultra-Endurance Event: 3040 Board# 326 May 30, 330 PM-500 PM. *Med. Sci. Sports Exerc.* 46:834.

[B8] Cohen J. (1988). *Statistical Power Analysis for the Behavioral Sciences*, 2nd Edn. Hillsdale: Lawrence Erlbaum Associates Inc.

[B9] Cona G. Cavazzana A. Paoli A. Marcolin G. Grainer A. Bisiacchi P. S. (2015). It’s a Matter of Mind! Cognitive Functioning Predicts the Athletic Performance in Ultra-Marathon Runners. *PLoS One* 10:e0132943. 10.1371/journal.pone.0132943 26172546 PMC4501764

[B10] Davies C. T. M. Thompson M. W. (1979). Aerobic performance of female marathon and male ultramarathon athletes. *Eur. J. Appl. Physiol. Occup. Physiol.* 41 233–245. 10.1007/BF00429740 499187

[B11] Der G. Deary I. J. (2006). Age and sex differences in reaction time in adulthood: results from the United Kingdom Health and Lifestyle Survey. *Psychol. Aging* 21 62–73. 10.1037/0882-7974.21.1.62 16594792

[B12] Doppelmayr M. M. Finkernagel H. Doppelmayr H. I. (2005). Changes in cognitive performance during a 216 kilometer, extreme endurance footrace: a descriptive and prospective study. *Percept. Mot. Skills* 100 473–487. 10.2466/pms.100.2.473-487 15974358

[B13] Durmer J. S. Dinges D. F. (2005). Neurocognitive consequences of sleep deprivation. *Semin. Neurol.* 25 117–129. 10.1055/s-2005-867080 15798944

[B14] Dykiert D. Der G. Starr J. M. Deary I. J. (2012). Sex differences in reaction time mean and intraindividual variability across the life span. *Dev. Psychol.* 48:1262. 10.1037/a0027550 22390656

[B15] Foulds H. J. Cote A. T. Phillips A. A. Charlesworth S. A. Bredin S. S. Burr J. F. (2014). Characterisation of baroreflex sensitivity of recreational ultra-endurance athletes. *Eur. J. Sport Sci.* 14 686–694. 10.1080/17461391.2014.884169 24601942

[B16] Garbisu-Hualde A. Santos-Concejero J. (2020). What are the limiting factors during an ultra-marathon? A systematic review of the scientific literature. *J. Hum. Kinet.* 72 129–139. 10.2478/hukin-2019-0102 32269654 PMC7126261

[B17] Gledhill N. Warburton D. Jamnik V. (1999). Haemoglobin, blood volume, cardiac function, and aerobic power. *Can. J. Appl. Physiol.* 24 54–65. 10.1139/h99-006 9916181

[B18] Halliwill J. R. Sieck D. C. Romero S. A. Buck T. M. Ely M. R. (2014). Blood pressure regulation X: what happens when the muscle pump is lost? Post-exercise hypotension and syncope. *Eur. J. Appl. Physiol.* 114 561–578. 10.1007/s00421-013-2761-1 24197081 PMC3944103

[B19] Hautala A. Tulppo M. P. Makikallio T. H. Laukkanen R. Nissila S. Huikuri H. V. (2001). Changes in cardiac autonomic regulation after prolonged maximal exercise. *Clin. Physiol.* 21 238–245. 10.1046/j.1365-2281.2001.00309.x 11318832

[B20] Hillman C. H. Erickson K. I. Kramer A. F. (2008). Be smart, exercise your heart: exercise effects on brain and cognition. *Nat. Rev. Neurosci.* 9 58–65. 10.1038/nrn2298 18094706

[B21] Hurdiel R. Pezé T. Daugherty J. Girard J. Poussel M. Poletti L. (2015). Combined effects of sleep deprivation and strenuous exercise on cognitive performances during The North Face^®^ Ultra Trail du Mont Blanc^®^ (UTMB^®^). *J. Sports Sci.* 33 670–674. 10.1080/02640414.2014.960883 25333827

[B22] Hurdiel R. Riedy S. M. Millet G. P. Mauvieux B. Pezé T. Elsworth-Edelsten C. (2018). Cognitive performance and self-reported sleepiness are modulated by time-of-day during a mountain ultramarathon. *Res. Sports Med.* 26 482–489. 10.1080/15438627.2018.1492401 29973086

[B23] Knechtle B. Nikolaidis P. T. (2018). Physiology and pathophysiology in ultra-marathon running. *Front. Physiol.* 9:634. 10.3389/fphys.2018.00634 29910741 PMC5992463

[B24] Krokosz D. Bidzan-Bluma I. Ratkowski W. Li K. Lipowski M. (2020). Changes of Mood and Cognitive Performance before and after a 100 km Nighttime Ultramarathon Run. *Int. J. Environ. Res. Public Health* 17:8400. 10.3390/ijerph17228400 33202782 PMC7697638

[B25] Lassen N. A. (1959). Cerebral blood flow and oxygen consumption in man. *Physiol. Rev.* 39 183–238. 10.1152/physrev.1959.39.2.183 13645234

[B26] Laughlin M. H. Davis M. J. Secher N. H. van Lieshout J. J. Arce-Esquivel A. A. Simmons G. H. (2011). Peripheral circulation. *Compr. Physiol.* 2 321–447. 10.1002/cphy.c100048 23728977

[B27] MacDonald J. R. (2002). Potential causes, mechanisms, and implications of post exercise hypotension. *J. Hum. Hypertens.* 16 225–236. 10.1038/sj.jhh.1001377 11967715

[B28] Martínez-Navarro I. Sanchez-Gomez J. M. Collado-Boira E. J. Hernando B. Panizo N. Hernando C. (2019). Cardiac Damage Biomarkers and Heart Rate Variability Following a 118-Km Mountain Race: relationship with Performance and Recovery. *J. Sports Sci. Med.* 18 615–622. 31827345 PMC6873135

[B29] Nikolaidis P. T. Knechtle B. (2018). Age of peak performance in 50-km ultramarathoners - is it older than in marathoners?. *Open Access J. Sports Med.* 9 37–45. 10.2147/OAJSM.S154816 29535560 PMC5840300

[B30] Ogoh S. Brothers R. M. Barnes Q. Eubank W. L. Hawkins M. N. Purkayastha S. (2005). The effect of changes in cardiac output on middle cerebral artery mean blood velocity at rest and during exercise. *J. Physiol.* 569 697–704. 10.1113/jphysiol.2005.095836 16210355 PMC1464249

[B31] Ogoh S. Tarumi T. (2019). Cerebral blood flow regulation and cognitive function: a role of arterial baroreflex function. *J. Physiol. Sci.* 69 813–823. 10.1007/s12576-019-00704-6 31444691 PMC10717347

[B32] Ramos-Campo D. J. Ávila-Gandía V. Alacid F. Soto-Méndez F. Alcaraz P. E. López-Román F. J. (2016). Muscle damage, physiological changes, and energy balance in ultra-endurance mountain-event athletes. *Appl. Physiol. Nutr. Metab.* 41 872–878. 10.1139/apnm-2016-0093 27447685

[B33] Robach P. Boisson R. C. Vincent L. Lundby C. Moutereau S. Gergelé L. (2014). Hemolysis induced by an extreme mountain ultra-marathon is not associated with a decrease in total red blood cell volume. *Scand. J. Med. Sci. Sports* 24 18–27. 10.1111/j.1600-0838.2012.01481.x 22672635

[B34] Roebuck G. S. Fitzgerald P. B. Urquhart D. M. Ng S.-K. Cicuttini F. M. Fitzgibbon B. M. (2018). The psychology of ultra-marathon runners: a systematic review. *Psychol. Sport Exerc.* 37 43–58. 10.1016/j.psychsport.2018.04.004

[B35] Romero S. A. Minson C. T. Halliwill J. R. (2017). The cardiovascular system after exercise. *J. Appl. Physiol.* 122 925–932. 10.1152/japplphysiol.00802.2016 28153943 PMC5407206

[B36] Saugel B. Cecconi M. Wagner J. Y. Reuter D. A. (2015). Noninvasive continuous cardiac output monitoring in perioperative and intensive care medicine. *Br. J. Anaesth.* 114 562–575. 10.1093/bja/aeu447 25596280

[B37] Scheer V. (2019). Participation Trends of Ultra Endurance Events. *Sports Med. Arthrosc. Rev.* 27 3–7. 10.1097/JSA.0000000000000198 30601393

[B38] Scott J. M. Esch B. T. Shave R. Warburton D. E. Gaze D. George K. (2009). Cardiovascular consequences of completing a 160-km ultramarathon. *Med. Sci. Sports Exerc.* 41 26–34. 10.1249/MSS.0b013e31818313ff 19092706

[B39] Simpson D. Post P. G. Young G. Jensen P. R. (2014). “It’s not about taking the easy road”: the experiences of ultramarathon runners. *Sport Psychol.* 28 176–185. 10.1123/tsp.2013-0064

[B40] Stanley J. Peake J. M. Buchheit M. (2013). Cardiac parasympathetic reactivation following exercise: implications for training prescription. *Sports Med.* 43 1259–1277. 10.1007/s40279-013-0083-4 23912805

[B41] Swart A. Constantinou D. (2020). The heart of an endurance athlete: impact of and recovery after an ultra-endurance event. *medRxiv* [Preprint]. 10.1101/2020.07.16.20155127

[B42] Tomoto T. Sugawara J. Hirasawa A. Imai T. Maeda S. Ogoh S. (2015). Impact of short-term training camp on arterial stiffness in endurance runners. *J. Physiol. Sci.* 65 445–449. 10.1007/s12576-015-0383-6 26037815 PMC10717420

[B43] Van Dongen H. P. Dinges D. F. (2005). Sleep, circadian rhythms, and psychomotor vigilance. *Clin. Sports Med.* 24 237–249. 10.1016/j.csm.2004.12.007 15892921

[B44] Van Lieshout J. J. Wieling W. Karemaker J. M. Secher N. H. (2003). Syncope, cerebral perfusion, and oxygenation. *J. Appl. Physiol.* 94 833–848. 10.1152/japplphysiol.00260.2002 12571122

[B45] Vlachopoulos C. Kardara D. Anastasakis A. Baou K. Terentes-Printzios D. Tousoulis D. (2010). Arterial stiffness and wave reflections in marathon runners. *Am. J. Hypertens.* 23 974–979. 10.1038/ajh.2010.99 20489686

[B46] Waldvogel K. J. Nikolaidis P. T. Di Gangi S. Rosemann T. Knechtle B. (2019). Women reduce the performance difference to men with increasing age in ultra-marathon running. *Int. J. Environ. Res. Public Health* 16:2377. 10.3390/ijerph16132377 31277399 PMC6651135

[B47] Warburton D. E. R. Jamnik V. K. Bredin S. S. D. Gledhill N. (2011). The Physical Activity Readiness Questionnaire for Everyone (PAR-Q+) and electronic Physical Activity Readiness Medical Examination (ePARmed-X+). *Health Fit. J. Can.* 4 3–23.

[B48] Warburton D. E. R. Nicol C. W. Bredin S. S. D. (2006). Health benefits of physical activity: the evidence. *Can. Med. Assoc. J.* 174 801–809. 10.1503/cmaj.051351 16534088 PMC1402378

[B49] Wollseiffen P. Schneider S. Martin L. A. Kerhervé H. A. Klein T. Solomon C. (2016). The effect of 6 h of running on brain activity, mood, and cognitive performance. *Exp. Brain Res.* 234 1829–1836. 10.1007/s00221-016-4587-7 26892883

[B50] Zimlichman R. Shargorodsky M. Boaz M. Duprez D. Rahn K.-H. Rizzoni D. (2005). Determination of arterial compliance using blood pressure waveform analysis with the CR-2000 system: reliability, repeatability, and establishment of normal values for healthy European population—the seven European sites study (SESS). *Am. J. Hypertens.* 18 65–71. 10.1016/j.amjhyper.2004.08.013 15691619

